# Pathway-based classification of genetic diseases

**DOI:** 10.1186/s13039-019-0418-4

**Published:** 2019-02-04

**Authors:** Ivan Y. Iourov, Svetlana G. Vorsanova, Yuri B. Yurov

**Affiliations:** 1 Mental Health Research Center, 117152 Moscow, Russia; 2 Veltischev Research and Clinical Institute for Pediatrics of the Pirogov Russian National Research Medical University, Ministry of Health of Russian Federation, 125412 Moscow, Russia; 3 Department of Medical Genetics, Russian Medical Academy of Continuous Professional Education, Moscow, 125993 Russia

**Keywords:** Chromosome, Disease, Genes, Classification, Genome, Pathway, Phenotype

## Abstract

**Background:**

In medical genetics, diseases are classified according to the nature (hypothetical nature) of the underlying genetic defect. The classification is “gene-centric” and “factor-centric”; a disease may be, thereby, designated as monogenic, oligogenic or polygenic/multifactorial. Chromosomal diseases/syndromes and abnormalities are generally considered apart from these designations due to distinctly different formation mechanisms and simultaneous encompassing from several to several hundreds of co-localized genes. These definitions are ubiquitously used and are perfectly suitable for human genetics issues in historical and academic perspective. However, recent achievements in systems biology have offered a possibility to explore the consequences of a genetic defect from genomic variations to molecular/cellular pathway alterations unique to a disease. Since pathogenetic mechanisms (pathways) are more influential on our understating of disease presentation and progression than genetic defects per se, a need for a disease classification reflecting both genetic causes and molecular/cellular mechanisms appears to exist. Here, we propose an extension to the common disease classification based on the underlying genetic defects, which focuses on disease-specific molecular pathways.

**Conclusion:**

The basic idea of our classification is to propose pathways as parameters for designating a genetic disease. To proceed, we have followed the tradition of using ancient Greek words and prefixes to create the terms for the pathway-based classification of genetic diseases. We have chosen the word “griphos” (γρῖφος), which simultaneously means “net” and “puzzle”, accurately symbolizing the term “pathway” currently used in molecular biology and medicine. Thus, diseases may be classified as monogryphic (single pathway is altered to result in a phenotype), digryphic (two pathways are altered to result in a phenotype), etc.; additionally, diseases may be designated as oligogryphic (several pathways are altered to result in a phenotype), polygryphic (numerous pathways or cascades of pathways are altered to result in a phenotype) and homeogryphic in cases of comorbid diseases resulted from shared pathway alterations. We suppose that classifying illness this way using both “gene-centric” and “pathway-centric” concepts is able to revolutionize current views on genetic diseases.

Classically, genetic point of view suggests diseases to be monogenic (digenic etc.), oligogenic, polygenic/multifactorial (complex) or chromosomal. This classification is based on either hypothetical or known nature of genetic defects underlying diseases [1, 2]. Actually, this classification is “gene-centric” and “factor-centric” leading to the dogma that genes and genetic-environmental interactions are the only parameters required to describe a disease with a genetic background. However, advances in genome research evidence that genetic diseases cannot be fully designated using genes and other regulatory elements [3, 4]. Furthermore, the concepts of designating genetic diseases (developed mainly for monogenic and multifactorial diseases) cannot be limited to specific genes or gene-gene interactions, but require extensive knowledge of gene-specific ontological properties and processes occurring at higher levels of causal interactions (i.e. systems biology hierarchy) [5, 6]. These requirements appear to be especially important for the description of chromosomal imbalances and disease-causing copy number variations (CNV). A problematic task is to classify diseases resulting from CNV, which are able to affect either single genes or several genes with random genomic localization producing extreme phenotypic heterogeneity. Consequently, manifestations of pathogenic CNV can be equal either to a monogenic disease or a chromosomal aberration [7–9]. On the other hand, monogenic diseases are not simple, exhibiting extreme variability in phenotypic manifestations and molecular/cellular mechanisms [6, 10]. These properties are also applicable to chromosomal syndromes (diseases) and abnormalities that usually encompass from several to several hundred of genes [11–13]. In total, the designation of genetic diseases as monogenic, polygenic/multifactorial or chromosomal superficially indicates possible or known genetic cause without reflecting the etiology, as a whole. Since etiology comprises the multilateral evaluation of how a disease can be classified, defined, and discovered [14], current classification of genetic diseases appears to require an update.

Although (cyto)genomic analysis is the permanent starting point for uncovering the mechanism and etiology of a disease, an indication of gene amount and a speculation about possible genetic-environmental interaction is certainly not enough for the disease designation at the present stage of development in the fields of (cyto)genomics and molecular (systems) medicine. The knowledge of the nature of genetic defects alone poorly defines the etiology of a disease. More precisely, mechanisms of phenotypic outcomes and molecular/cellular pathways to disease remain obscure without a presentation of additional etiologic aspects. Particularly, addressing numerical and structural abnormalities of chromosomes using “gene-centric” concepts is usually confined to the determination of amount of affected genes. However, it is possible that a limited number of genes within the rearranged chromosomal region are intrinsically involved in the clinical outcome. CNV or mutations in different genes attributed to the same pathway may have clinical outcomes similar to chromosome rearrangements or vice versa [7, 13, 15]. For instance, our previous studies of mutation-negative cases of a monogenic disease (Rett syndrome) have shown that the disease can be caused by subchromosome rearrangements (microdeletions), as well [16]. Even though it is the same disease from a clinical point of view, one has to differ between “monogenic” and “chromosomal” Rett syndrome. Single gene mutations are able to produce chromosomal/genomic instability, which is the underlying cause of the clinical outcome [17, 18]. Genome/chromosome instability syndromes (monogenic syndromes) usually exhibit severe manifestations inasmuch as numerous molecular and cellular pathways are altered due to a mutation in a regulatory gene. Thus, it is quite strange that diseases associated with a single pathway defect (e.g. single enzymatic defect) are attributed to the same category as diseases associated with an extensive cascade of abnormal molecular and cellular events. More importantly, these genetic conditions can be merely defined as monogenic, because the underlying cause of the disease manifestations is chromosome/genome instability representing the simultaneous presence of multiple DNA sequence mutations and/or chromosome abnormalities. Accordingly, it is necessary to highlight another problem in classifying diseases caused by genomic variations, which derives from the presence of multiple rearrangements in an individual genome possessing cumulative effect and producing interindividual phenotypic heterogeneity and intercellular genetic variability. The latter underlies numerous complex (polygenic or multifactorial) diseases (i.e. cancer, neurodegenerative and neuropsychiatric disorders) and can only be properly assessed by network-based/pathway-based analyses [19, 20]. Multilateral genomic instability in cancer has a wide range of origins (genetic and environmental). However, regardless of the nature of “initial” genetic defect (monogenic or chromosomal) or starting point of clonal somatic genome evolution, almost all cancers are likely to result from alterations to shared and specific pathways [21, 22]. Pathway-based analysis provides more precise cancer classification than focusing on multitude of different genomic and chromosomal variations in malignant cell populations [23]. Genetic architecture of other complex diseases (e.g. neuropsychiatric and neurodegenerative disorders) seems to be alike [24]. It is highly probable that neuropsychiatric disorders are the result of complex interactions and cascades of single gene mutations, chromosomal abnormalities, somatic mosaicism/genome instability and genetic-environmental interactions [25]. In autism, shared and specific molecular/cellular pathways are disabled by a wide spectrum of genomic alterations. Similarly to cancer, pathway-based analysis determines converging molecular pathways to the disease [26, 27]. Despite a stricter clinical definition, Alzheimer’s disease also exhibits extreme variability of the underlying causes. Additionally, Alzheimer’s disease is associated with somatic mosaicism and chromosome (genome) instability confined to the diseased brain originating from disruptions of cell cycle checkpoint, mitotic signaling and DNA replication pathways [28–30]. Since brain pathology featuring Alzheimer’s disease is able to be produced either by single-gene mutations or by environmental factors, it is recommended to designate the disease using pathway-based approaches to unravel the pathogenetic mechanisms [31]. Finally, mutations of genes integrated in a single pathway (functionally related genes) with similar clinical outcomes seem to be the most probable explanation for comorbidity and blurred distinction between monogenic and complex forms [32, 33]. It is noteworthy that pathway-based (network-based) approaches to molecular etiology of complex disease comorbidities shed light on the mechanisms and fascinate the development of targeted therapeutic strategies [34]. In summary, pathway-based analysis of genomic variations is able to add significant information to the data on DNA sequence changes [19–23, 25–27, 30, 33, 34]. Therefore, a classification for genetic diseases is likely to benefit from the knowledge of pathways altered to result in specific phenotype.

The idea of human disease classification using pathway-based approaches to the molecular and cellular mechanisms has marked the beginning of the postgenomic era [35]. Focusing on uncovering underlying disease mechanisms using high-resolution genomic data, pathway-based analysis has provided numerous discoveries in the field of molecular medicine. As a result, it is possible to associate a disease not only with specific genomic variations, but also with specific cellular phenotypes and biomarkers or, in other words, with molecular pathways unique to a disease [36–38]. To understand functional consequence of genome changes, genomic variation has to be assessed by systems biology approaches to unveil molecular pathways [39]. These approaches are also applicable for chromosome abnormalities resulting in narrowing genotype-phenotype correlations and uncovering intrinsic causal interactions at genomic, transcriptomic, proteomic, and metabolomic levels [40]. Moreover, it has been consistently shown that the etiology of complex diseases may be unraveled almost exclusively by pathway-based analysis of genomic data [41, 42]. In this context, it is to note that complex (multiple) phenotypes resulted from genomic variations are better classified using pathway-based multivariate analysis [43]. Finally, pathway-based analysis, providing the knowledge about the number and extent of alterations to the molecular and cellular pathways, is able to form theoretical and even empirical basis for the treatment of presumably incurable genetic conditions (i.e. chromosome instability syndromes, structural chromosome abnormalities, complex diseases) [44–46]. As one can see, pathways are as important as genes for understanding disease etiology.

The “gene-centric” model established during the last three decades has become almost non-competitive for classification of genetic diseases [3, 4]. From the theoretical point of view, a model’s ability to describe phenomena should come at the expense as our knowledge deepens. To provide actual scientific explanation of phenomena, collecting (combining) models seems to be the way to increase the explanatory power [47]. Interestingly, theoretic analysis of biological explanations (definitions of biomedical phenomena) accentuates the role of pathway concept in explaining multicausal relationships between components of a biological system [48]. Therefore, an extension of “gene-centric” model for classifying genetic diseases by combining it with “pathway-centric” model is able to extend the explanatory power. Eventually, theoretical and empirical considerations indicate that classifying genetic diseases using the number of altered pathways in addition to designating hypothetical or known nature of the genetic defects has to be given a chance to be developed.

Following the tradition of creating biomedical terms using ancient Greek words and prefixes, we have proposed to use the word “griphos” (γρῖφος) for making disease designations. The ancient Greek word “griphos” simultaneously means “net” and “puzzle” and adequately symbolizes the term “pathway” in the context of molecular biology and medicine. Combining the word “griphos” and Greek prefixes, following disease designations are suggested: monogryphic — single pathway is altered to result in a phenotype; digryphic, trigriphic, etc.… — two, three, etc. pathways are altered to result in a phenotype; oligogryphic — several pathways are altered to result in a phenotype; polygryphic — numerous pathways or cascades of pathways are altered to result in a phenotype; homeogryphic — shared pathway alterations can result in comorbid diseases.

Natural limitations of the pathway-based classification are associated with a possible lack of knowledge about specific pathways altered in a given disease. However, this is also the case for genetic variants (defects), which are attempted to be associated with complex diseases. Poor reproducibility of findings in genetic studies of complex diseases indicates that pathway-based classification focused on candidate processes might be a solution for etiologic analysis of multifactorial disorders [19, 20, 27, 40]. Additional limitation of the classification can result from difficulties of pathway definitions. The hierarchy of pathways is not precisely determined suggesting the existence of general pathways encompassing less sophisticated ones. In this case, it is hard to indicate whether the disease is associated with a single general pathway (monogryphic) or numerous pathways (polygryphic) of “lower hierarchical levels” are implicated in the etiology. It is highly likely that forthcoming studies on pathway hierarchy may give a solution to this problem.

We outline below some examples of using the proposed classification extension:

- A disease caused by a single gene mutation and associated with a phenotype produced by an alteration to a single pathway (i.e. monogenic metabolic diseases) would be defined as “monogenic-monogryphic disease”.
- A disease caused by a single gene mutation resulting in a pathogenetic cascade altering numerous pathways (i.e. chromatin remodeling diseases, chromosome instability syndromes) would be defined as “monogenic-polygryphic disease”.
- Chromosomal abnormalities altering several pathways would be defined as “chromosomal-oligogryphic diseases”.
- A chromosomal syndrome resulting in a rearrangement of a chromosomal locus containing several genes, two of which alter two pathways specific for the syndrome manifestations would be defined as “chromosomal-digryphic disease”.
- Comorbid diseases, which are caused by mutations in different genes involved in a single pathway, would be defined as “monogenic-homeogryphic diseases”.
- Cases of alterations to shared pathways due to the complex interaction between genetic and environmental factors would be defined as “polygenic or multifactorial-homeogryphic diseases”.
- A complex disease caused by an alteration to a specific pathway, which may occur due to a variety of single-gene mutations, chromosome abnormalities and genetic-environmental interactions would be defined as “polygenic or multifactorial-monogryphic disease”.

To show the applicability of the disease classification, we provide more precise examples:

(i) phenylketonuria (a disorder caused by mutations in the *PAH* gene, encoding phenylalanine hydroxylase catalyzing a reaction of the hydroxylation of phenylalanine to tyrosine) — monogenic-monogryphic disease;
(ii) Rett syndrome (a disorder caused by genetic defects in *MECP2*, a gene involved in several pathways mainly regulating genome activity) — monogenic-polygryphic disease;
(iii) ataxia-telangiectasia (a chromosome instability syndrome caused by genetic defects in *ATM*, a gene involved in a multitude of pathways regulating genome stability maintenance, cell cycle, programed cell death etc.) — monogenic-polygryphic disease;
(iv) familial Alzheimer’s disease (rare familial cases of Alzheimer’s disease, mainly considered as multifactorial, are caused by mutations in single genes implicated in multiple pathways) — monogenic-polygryphic disease;
(v) sporadic Alzheimer’s disease (a multifactorial disorder associated with a variety of genetic defects resulting in alterations to multiple pathways) — multifactorial-polygryphic disease;
(vi) Williams syndrome (a chromosomal syndrome caused by microdeletions at 7q11.23 leading to a disbalance of 20–30 genes affecting several pathways) — chromosomal-oligogryphic disease;
(vii) A unique case of chromosomal microdeletion at 3p22.1p21.31 resulting in alterations of two pathways (for more details, see [45]) — chromosomal-digryphic disease.

The present extension to the common disease classification is not suggested to substitute the ultimately accepted designations of human diseases (i.e. monogenic, poygenic/multifactorial/complex and chromosomal). Indeed, an addition to the indication of the nature (hypothetical nature) of the underlying genetic defect highlighting the disease-specific molecular pathway appears to be required both for medical studies and for academic research. To this end, we do hope that this classification extension using both “gene-centric” and “pathway-centric” concepts may revolutionize current views on genetic diseases.

## Abbreviations

CNV: Copy number variations
